# Corneal Biomechanical Findings in Contact Lens Induced Corneal Warpage

**DOI:** 10.1155/2016/5603763

**Published:** 2016-09-04

**Authors:** Fateme Alipour, Mojgan Letafatnejad, Amir Hooshang Beheshtnejad, Seyed-Farzad Mohammadi, Seyed Reza Ghaffary, Narges Hassanpoor, Mehdi Yaseri

**Affiliations:** ^1^Eye Research Center, Farabi Eye Hospital, Tehran University of Medical Sciences, Tehran, Iran; ^2^Tehran University of Medical Sciences, Tehran, Iran; ^3^Eye Research Center and Department of Epidemiology and Biostatistics, School of Public Health, Tehran University of Medical Sciences, Tehran, Iran

## Abstract

*Purpose*. To evaluate the difference in biomechanical properties between contact lens induced corneal warpage and normal and keratoconic eyes.* Method*. Prospective observational case control study, where 94 eyes of 47 warpage suspicious and 46 eyes of 23 keratoconic patients were included. Warpage suspected cases were followed until a definite diagnosis was made (warpage, normal, or keratoconus).* Results*. 44 eyes of 22 patients had contact lens related corneal warpage. 46 eyes of 23 people were diagnosed as nonwarpage normal eyes. 46 eyes of 23 known keratoconus patients were included for comparison. The mean age of the participants was 23.8 ± 3.8 years, and 66.2% of the subjects were female. The demographic and refractive data were not different between warpage and normal groups but were different in the keratoconus group. The biomechanical properties (corneal hysteresis or CH and corneal resistance factor or CRF) were different with the highest value in the warpage group followed by normal and keratoconus groups. CRF was 10.08 ± 1.75, 9.23 ± 1.22, and 7.38 ± 2.14 and CH was 10.21 ± 1.57, 9.59 ± 1.21, and 8.69 ± 2.34 in the warpage, normal, and keratoconus groups, respectively.* Conclusion*. Corneal biomechanics may be different in people who develop contact lens induced warpage.

## 1. Introduction

Contact lens induced corneal warpage is occasionally seen in people who regularly wear contact lenses [1, 2]. Considering the high prevalence of the contact lens use among refractive surgery candidates, the clinical picture could be especially confusing in the setting of refractive surgery when the condition can mimic pathologies like keratoconus which is commonly considered an absolute contraindication to refractive surgery [3]. The usual suggested “off contact lens” waiting period in most refractive surgery clinics is about 2 weeks [4], which is not necessarily enough for resolving the warpage induced by the long-term use of contact lenses. This waiting period (which may be even more than 2 months) [1, 5, 6] is not acceptable for many of the people who are accustomed to a spectacle-free lifestyle and have planned to have refractive surgery at a specific time.

Apart from serial examinations and follow-ups which are the definite way to differentiate contact lens induced corneal warpage [7, 8], other features like the corneal topography and corneal thickness pattern [9] have been proposed to help with the differentiation of this condition from keratoconus. Considering the major role of corneal biomechanical properties in the pathogenesis of keratoconus, there may be a difference in the corneal biomechanical parameters between true keratoconic and corneal warpage in contact lens using patients in theory.

In this study, we evaluated the biomechanical changes (using the Ocular Response Analyzer) in patients presenting with corneal warpage and compared these changes with biomechanical findings in patients with keratoconus. The corneal biomechanical parameters, corneal hysteresis (CH) and corneal resistance factor (CRF), are commonly used for this purpose.

The corneal biomechanical parameters (CH and CRF) can be assessed by the Ocular Response Analyzer (ORA, Reichert, Corp., Buffalo, NY). ORA measures the corneal response to a rapid air pulse. Two measurements of the intraocular pressure at the first applanation (*P*1) and the second applanation (*P*2) when the cornea flattens again as the air pressure falls are made. It has been found that the second applanation occurs at a lower pressure than the first applanation. The difference between the two pressures has been termed CH and represents a function related to the viscoelastic properties of the cornea [10]. In addition to CH, the ORA measures CRF, which is derived from the formula (*P*1 − *kP*2), where *k* is a constant empirically determined so that CRF is more strongly associated with the central corneal thickness (CCT) than CH [11, 12].

## 2. Methods

### 2.1. Prospective Observational Case/Control Study

From the refractive surgery candidates of the Farabi Eye Hospital Refractive Surgery Clinic, 94 eyes of 47 people who were suspicious for corneal warpage, based on the corneal topographic pattern, were included in the study in the case group. For the control group, 46 eyes of 23 known keratoconic patients who never wore contact lenses were included. These control patients were selected from the Cornea Clinic of Farabi Eye Hospital.

Patients with any form of corneal scarring were excluded from the study. Abnormal topography in the corneal warpage group was defined as central irregular astigmatism, loss of radial symmetry, and reversal of the normal topographic pattern of progressive flattening of the corneal contour from the center to the periphery [13]. The Pentacam (Oculus Optikgeräte GmbH, Wetzlar, Germany) or Orbscan (Bausch & Lomb, Rochester, NY) were used for topographic measurements. The corneal biomechanical parameters (CH and CRF) were assessed by Ocular Response Analyzer (ORA, Reichert, Corp., Buffalo, NY).

In the case group, the patients were asked not to wear their contact lenses for 2 to 4 more weeks and return to the clinic for examinations. Refraction, corneal imaging (Orbscan II or Pentacam), and ORA measurements (CH and CRF) were repeated. At this stage, based on the changes in the topographic pattern, the patients were diagnosed as follows:

1. Contact lens induced corneal warpage—complete resolution.
2. Possible contact lens induced corneal warpage—incomplete resolution.
3. Keratoconus.

The criteria for stabilization were defined as (1) manifest refraction changes within 0.50 D, (2) keratometry changes within 0.50 D, and (3) a normal corneal topography pattern [14].

Those with suspicious incomplete warpage resolution were requested to wait for 2–4 more weeks and all the abovementioned examinations were repeated at each follow-up visit. The ORA measures were repeated in each follow-up visit. To reduce variability due to diurnal variations in the corneal thickness [15] and CH [10], all evaluations were performed between 11:00 and 14:00 pm. At least three acceptable ORA measurements were recorded for each patient and the mean value was considered for data entry.

The final categories were based on the consensus of 3 cornea subspecialists (AH.B., F.A., and SF.M.), reviewing all the records as

1. Contact lens induced corneal warpage.
2. Nonwarpage normal.
3. Keratoconus.

Diagnosis of keratoconus was made based on a combination of clinical signs like the presence of the scissor reflex on retinoscopy and biomicroscopic findings such as stromal thinning, conical protrusion, Fleischer's ring, Vogt's striae, and enlarged corneal nerves and topographic findings consistent with keratoconus like an asymmetric bow tie pattern (AB) or skewed radial axes (SRAX). Nonwarpage normal was defined as a stable pattern and refraction not compatible with keratoconus, and contact lens induced corneal warpage was defined as a refraction and topographic pattern compatible with warpage returning to normal.

The demographic data, final CRF, and final CH were compared between these groups. Changes in CH and CRF were also evaluated in the case group.

The protocol of the study adhered to the tenets of the Declaration of Helsinki and informed consent was obtained from all participants.

## 3. Statistical Analysis

Descriptive statistics were used to evaluate the distribution of the data. The normality of the data was tested with Kolmogorov-Smirnov/Shapiro-Wilk test. We used the paired *t*-test to assess the changes within groups (corneal warpage). As for the difference in the baseline pachymetry, Analysis of Covariance (ANOVA) with adjustment for corneal thickness was used to evaluate the difference between the groups. Considering the possible correlation of the results in two eyes, we applied the GEE analysis. We used Bonferroni method to adjust for multiple comparisons. *P* values less than 0.05 were considered statistically significant. All statistical analyses were performed with SPSS software (IBM SPSS Statistics for Windows, Version 22.0., Armonk, NY: IBM Corp.). *P* values less than 0.05 were considered significant.

**Figure 1:**
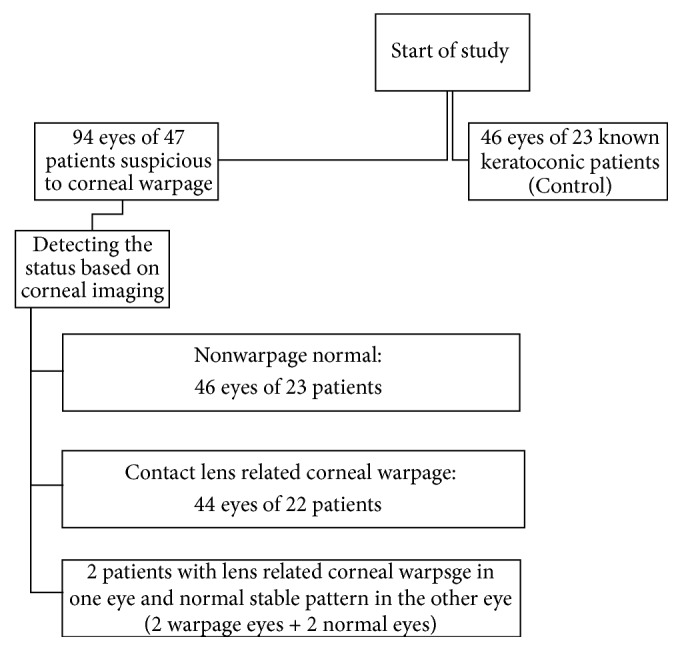
Study subjects.

Finally, 44 eyes of 22 patients were found to have contact lens related corneal warpage. Frothy-six eyes of 23 patients were diagnosed as nonwarpage normal eyes. Two patients had warpage in only one eye and a nonwarpage normal stable pattern in the other eye. Frothy-six eyes of 23 known keratoconus patients were included for comparison (Figure 1).

Demographic and refractive data are shown in Table 1. There was a significant difference between the final diagnosis categories regarding the age (*P* < 0.001), sex (*P* < 0.001), astigmatism (*P* < 0.001), and the thinnest point (*P* = 0.004). According to the post hoc analysis (adjusted for multiple comparisons by the Bonferroni method), the keratoconus group had a significant difference in age with nonwarpage normal and warpage groups (both *P* < 0.001) but there was no difference between the nonwarpage normal and warpage group (*P* > 0.99). Gender difference was significant between keratoconus group and the two other groups (the warpage and the normal nonwarpage groups) with higher male to female ratio in the keratoconus group, (both *P* < 0.001) but there was no difference between the nonwarpage normal and warpage group (*P* = 0.868). The same was true for the difference in astigmatism (*P* < 0.001 for both keratoconus group and nonwarpage normal and warpage and *P* > 0.99 for the normal group versus the warpage group) and the thinnest point (*P* = 0.003 for keratoconus versus nonwarpage normal, *P* = 0.004 for keratoconus versus warpage, and *P* > 0.99 for normal versus warpage).

Corneal biomechanical indices are shown in Tables 2 and 3. CH, CRF, and CCT were significantly different between groups. The Bonferroni method was used for multiple comparisons. Significant values are bolded in Table 3.

## 4. Discussion

Analysis of the demographic data showed no significant differences in age, sex, and refraction between patients who suffered from warpage and normal participants without warpage. However, the participants in the keratoconus group were younger with a higher male to female ratio, thinner corneas, higher refractive errors, and lower best corrected visual acuity. Although this finding could be predicted because of our selection method which was to include documented keratoconus patients who never used contact lenses in our control group, it might have resulted in some biases in our findings [16].

In our study, the mean CH and CRF were 9.59 ± 1.21 and 9.23 ± 1.22 in the normal group, respectively, which was lower than the results of a study by Sedaghat et al. that was conducted on a normal population from the north eastern part of Iran without considering the history of the contact lens use (the mean CH and CRF for all eyes were 9.9 ± 1.4 and 10.1 ± 1.6 mmHg, resp.) [17]. This may be either due to changes induced by the long-time contact lens wearing in our population, even those who had stable topographic and refractive results after discontinuation of the contact lens use, or due to different study populations. Other studies have reported even higher CH and CRF values in the normal population [18, 19].

Interestingly, despite the fact that our first theory was “those who develop warpage are possibly more similar to keratoconus patients,” we found a significant difference in CH and CRF between participants with documented corneal warpage, normal participants (no warpage), and keratoconus patients; the highest values were seen in the warpage group. These findings show that there might be a structural difference in the cornea of the people who develop corneal warpage. Clinicians may be able to use this difference for predicting the development of corneal warpage in those who wish to use contact lenses. Another possible clinical implication of these findings is shortening the waiting time for the resolution of possible corneal warpage.

A study on changes in the corneal biomechanical indexes after wearing orthokeratology contact lenses reported a decrease in CRF that correlated with the duration of the contact lens use [20].

The idea of evaluating corneal biomechanical properties for differentiating contact lens induced corneal warpage from keratoconus is a novel idea. The results of this study showed that there might be a potential application for detecting the subjects who are prone to warpage. Further studies are required on the corneal structure and histology of these three groups, with including one normal group with no history of the contact lens use, to prove our findings and find biological explanations for these differences. It is also recommended to use more advanced methods for the measurement of corneal biomechanical properties.

## Figures and Tables

**Table 1:** Demographic data according to the final diagnosis.

Parameter		Total	Group			*P*
			Keratoconus	Normal	Warpage	
Age	Mean ± SD	23.8 ± 3.8	21.3 ± 2.9	25.5 ± 4.3	25 ± 2.8	<0.001^†^

Sex	Female	43 (66.2%)	8 (34.8%)	16 (80.0%)	19 (86.4%)	<0.001^*∗*^
	Male	22 (33.8%)	15 (65.2%)	4 (20.0%)	3 (13.6%)	

Sphere	Mean ± SD	−3.39 ± 2.47	−2.98 ± 3.83	−3.55 ± 1.35	−3.55 ± 1.99	0.521^§^

Astigmatism	Mean ± SD	−1.4 ± 2.31	−3.42 ± 3.14	−0.58 ± 1.35	−0.66 ± 1.13	<0.001^§^

Spherical equivalent	Mean ± SD	−4.11 ± 2.73	−4.79 ± 4.31	−3.84 ± 1.33	−3.88 ± 2.19	0.847^§^

BCVA (decimal)	Mean ± SD	0.95 ± 0.6	0.86 ± 1.11	0.99 ± 0.05	0.99 ± 0.04	0.775^§^

Thinnest	Mean ± SD	534 ± 51	469 ± 60	535 ± 27	552 ± 50	0.004^§^

^†^Based on analysis of variance (ANOVA).

^*∗*^Based on chi-square test.

^§^Based on GEE analysis.

**Table 2:** Differences in the corneal biomechanical indexes according to the baseline diagnosis.

		Total	Group		Diff	95% CI		*P* ^§^
			Warpage	Keratoconus		Lower	Upper	
CRF	Mean ± SD	9.06 ± 1.96	9.57 ± 1.59	7.38 ± 2.14	2.03	0.95	3.11	<0.001
	Median (range)	9 (3.75 to 14.13)	9.25 (6.4 to 14.13)	7.39 (3.75 to 13.3)				

CH	Mean ± SD	9.55 ± 1.76	9.81 ± 1.46	8.69 ± 2.34	1.01	0.02	2.03	0.056
	Median (range)	9.25 (4.05 to 15.56)	9.6 (6.6 to 13.65)	8.49 (4.05 to 15.56)				

CCT	Mean ± SD	542 ± 47	551 ± 39	480 ± 60	75	29	121	0.001
	Median (range)	548 (423 to 639)	549 (453 to 639)	463 (423 to 600)				

^§^Based on GEE analysis.

**Table 3:** Corneal biomechanical indexes according to the final diagnosis.

		Keratoconus	Nonwarpage normal	Warpage	*P* ^§^	*P*1	*P*2	*P*3
CRF	Mean ± SD	7.38 ± 2.14	9.23 ± 1.22	10.08 ± 1.75	<**0.001**	**0.002**	<**0.001**	0.066
	Median (range)	7.39 (3.75 to 13.3)	9.15 (6.4 to 13)	9.63 (7.3 to 14.13)				

CH	Mean ± SD	8.69 ± 2.34	9.59 ± 1.21	10.21 ± 1.57	**0.028**	0.158	**0.013**	0.069
	Median (range)	8.49 (4.05 to 15.56)	9.5 (6.6 to 13.2)	10.14 (7.65 to 13.65)				

CCT	Mean ± SD	480 ± 60	542 ± 26	562 ± 48	**0.005**	**0.002**	**0.001**	0.83
	Median (range)	463 (423 to 600)	548 (489 to 593)	556 (453 to 639)				

Significant values are shown bolded.

^§^Based on GEE analysis.

*P*1: Comparison of keratoconus versus normal adjusted for multiple comparison based on Bonferroni method.

*P*2: Comparison of keratoconus versus warpage adjusted for multiple comparison based on Bonferroni method.

*P*3: Comparison of normal versus warpage adjusted for multiple comparison based on Bonferroni method.
